# Right Ventricle Perforation Post Pacemaker Insertion Complicated with Cardiac Tamponade

**DOI:** 10.7759/cureus.2266

**Published:** 2018-03-04

**Authors:** Muhammad Khalid, Ghulam Murtaza, Muhammad Talha Ayub, Vijay Ramu, Timir Paul

**Affiliations:** 1 Department of Internal Medicine, East Tennessee State University; 2 Internal Medicine, John H Stroger J. Hospital of Cook County; 3 Cardiology, Division of Cardiology, East Tennessee State University

**Keywords:** cardiac tamponade, permanent pacemaker, right ventricular

## Abstract

Pacemaker-lead-associated right ventricular perforation is a life-threatening complication. Acute perforation usually presents within 24 hours. Patients with lead perforation are often asymptomatic but fatal complications like hemopericardium, leading to cardiac tamponade and death, are reported. Diagnosis is based on chest x-ray, computed tomography (CT) scan, and echocardiography. The management of the lead perforation is based on clinical presentation. Extraction is avoided in cases of chronic asymptomatic lead perforations because of the associated complications. Urgent intervention is needed in hemodynamically unstable patients with pericardial effusion or cardiac tamponade physiology.

## Introduction

Cardiac pacemaker permanent devices are widely used in the management of arrhythmias. Right heart perforation following pacemaker or implantable cardioverter defibrillator is a rare life-threatening complication that can cause the rapid development of cardiac tamponade, needing aggressive supportive measures and pericardiocentesis. We present a case of a 66-year-old female with cardiogenic shock secondary to cardiac tamponade from right ventricular perforation within 24 hours of pacemaker implantation.

## Case presentation

**Figure 1:**
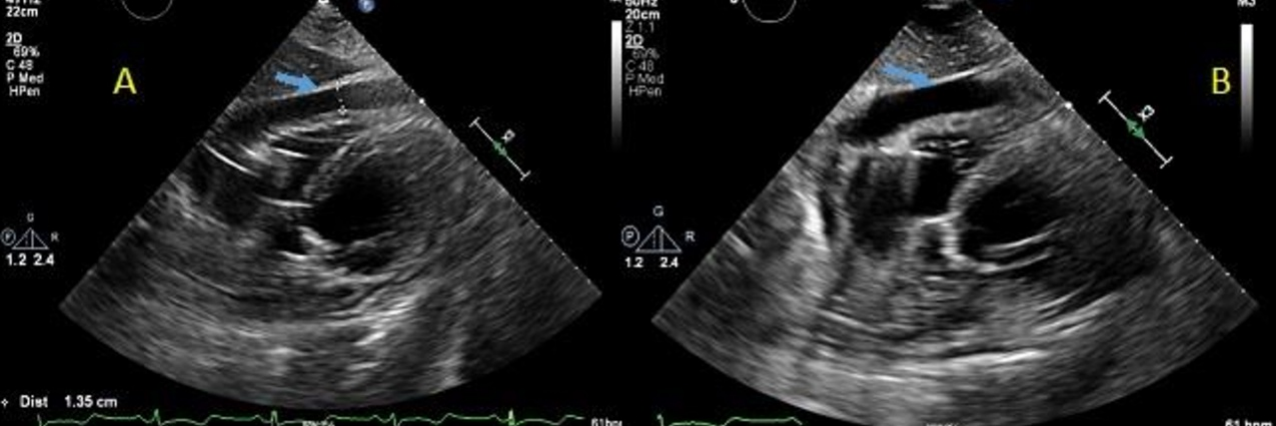
Echocardiogram showing pericardial effusion causing right atrial compression in diastole

A 66-year old female with a past medical history of hypertension, hyperlipidemia, and hypothyroidism was admitted for the progressively worsening shortness of breath and fatigue from the last one week. The patient reported increased somnolence and intermittent syncopal episodes for the last one year. On admission, the physical exam showed a blood pressure of 104/86 mm Hg, a temperature of 96.9 °F, a respiratory rate of 19 per min, a pulse of 110 beats per min, and normal oxygen saturation was 97%. The cardiopulmonary examination was significant for an irregular rhythm with a grade III/VI pansystolic murmur at the left sternal border and bibasilar crackles with decreased breath sounds. Laboratory workup showed potassium 3.4, brain natriuretic peptide 4741, troponins <0.03, and white blood cell count 9.9. The chest x-ray showed a left-sided pleural effusion. Electrocardiography (ECG) showed atrial fibrillation with a rapid ventricular response. In the emergency department, the patient had witnessed an episode of syncope. The telemetry monitor showed three ventricular pauses of 8-9 seconds during that syncopal episode. The transthoracic echocardiogram (TTE) showed a new reduction in left ventricular systolic function; ejection fraction (EF) of 15-20%; global hypokinesis; and moderate mitral regurgitation as compared to an EF of 60-65% on previous TTE. The patient had this new onset decompensated heart failure possibly secondary to tachycardia-induced cardiomyopathy. The patient was evaluated by an electrophysiologist and had a dual chamber permanent pacemaker (PPM) placement due to significant ventricular pauses with a recommendation to hold on to the atrioventricular nodal (AV) blocker and started on amiodarone as a temporary antiarrhythmic. The patient had a spontaneous conversion to normal sinus rhythm. The post pacemaker chest x-ray showed appropriate lead placement with no pneumothorax. Overnight, the patient had an acute onset of chest and neck pain with the associated shortness of breath. The patient became hemodynamically unstable with a systolic blood pressure of 56/76 mm Hg, pulse 70 beats per minute, and respiratory rate 24 per min, nonresponsive to fluid bolus and requiring vasopressor support. A stat bedside echocardiogram showed a medium-sized circumferential pericardial effusion with early tamponade physiology (Figure 1 A-B).

The patient responded well to levophed and was gradually weaned off the next day. During the hospital course, the patient remained hemodynamically stable with no more ventricular pauses. A repeat TTE showed stable pericardial effusion. The patient had a left heart catheterization later, which demonstrated normal coronaries, ejection fraction of 15%-20%, and normal left ventricle filling pressure. Anticoagulation was started 48 hours post device placement. Cardiology recommended the continuation of amiodarone. The patient was also started on digoxin and the AV nodal blocker was avoided because of hypotension. The pacemaker pocket site examination one week later showed no erythema. The patient was later started on metoprolol and spironolactone on discharge for the optimal management of non-ischemic cardiomyopathy. 

## Discussion

Cardiac pacemaker devices are used for temporary as well as permanent management options for various cardiac conduction abnormalities and arrhythmias. The various device-related complications are divided into three categories: acute within 24 hours, subacute up to one month, and chronic/delayed after one month. The most common, reported complications are pneumothorax, lead malposition, myocardial perforation, displacement or fracture due to manipulation by the patient (Twiddler’s syndrome), infectious complications related to pacemaker pocket infection or endocarditis, lead sensing or pacing failure, lead erosion, and subclavian thrombosis [1-2]. Right ventricular perforation is an uncommon but life-threatening complication of pacemakers (PMs) and implantable cardioverter defibrillator (ICD) placement, with an incidence of 0.1%-0.8% and 0.6%-5.2%, respectively [3]. The right ventricular apex has been reported the most common site of perforation due to the thin wall [4].

Clinical presentation is diverse. Patients can remain asymptomatic or present with chest pain, dyspnea, syncope, abdominal pain, hiccups, and cardiogenic shock due to hemopericardium with the resultant cardiac tamponade, pleural effusion, and pacing and sensing failure [5].

Different factors associated with pacemaker perforation include temporary leads, steroid use, old age, low body mass index, active fixation leads, and concomitant anticoagulation [6]. Right ventricular perforation is less commonly seen with right ventricular hypertrophy. Right ventricular systolic pressure > 35 mm Hg is a protective factor against perforation [7].

Different modalities are used to diagnose device-related complications. The chest x-ray and echocardiogram are non-invasive and easily available bedside modalities to diagnose pacemaker complications, including pericardial or pleural effusion and lead displacement. A computed tomography (CT) scan of the chest can be used to confirm pericardial or pleural effusion and lead position or displacement [8]. Device interrogation is very important, as perforation usually result in pacemaker sensing and pacing failure; however, normal pacemaker function does not rule out pacemaker perforation [9].

The management of pacemaker lead perforation depends upon the clinical presentation, pericardial effusion, and hemodynamic status [10]. Emergent surgical management is the best treatment in cases of rapidly progressive pericardial effusion with cardiac tamponade, hemodynamically unstable patients, and large pleural effusions with respiratory compromise. The management of lead perforation in hemodynamically stable patients is debatable and usually done with percutaneous extraction under close echocardiographic or fluoroscopic monitoring with surgery backup. Lead extraction should be followed by the placement of a new lead in a different location, preferably in the right ventricular outflow tract or the interventricular septum.

## Conclusions

Right ventricular perforation post pacemaker insertion is a rare complication that sometimes could be life-threatening. A post-pacemaker or ICD implantation chest x-ray is very useful to look for immediate complications. The management of pacemaker lead perforation is debatable. Usually, immediate intervention is needed in hemodynamically unstable patients, pericardial effusion with cardiac tamponade, and large pleural effusions.
